# Glutamine Transport and Mitochondrial Metabolism in Cancer Cell Growth

**DOI:** 10.3389/fonc.2017.00306

**Published:** 2017-12-11

**Authors:** Mariafrancesca Scalise, Lorena Pochini, Michele Galluccio, Lara Console, Cesare Indiveri

**Affiliations:** ^1^Department DiBEST (Biologia, Ecologia, Scienze della Terra), Unit of Biochemistry and Molecular Biotechnology, University of Calabria, Arcavacata di Rende, Italy; ^2^CNR Institute of Biomembranes, Bioenergetics and Molecular Biotechnology, Bari, Italy

**Keywords:** tumors, mitochondria, metabolism, proteoliposome, plasma membrane, drug design

## Abstract

The concept that cancer is a metabolic disease is now well acknowledged: many cancer cell types rely mostly on glucose and some amino acids, especially glutamine for energy supply. These findings were corroborated by overexpression of plasma membrane nutrient transporters, such as the glucose transporters (GLUTs) and some amino acid transporters such as ASCT2, LAT1, and ATB^0,+^, which became promising targets for pharmacological intervention. On the basis of their sodium-dependent transport modes, ASCT2 and ATB0^+^ have the capacity to sustain glutamine need of cancer cells; while LAT1, which is sodium independent will have the role of providing cancer cells with some amino acids with plausible signaling roles. According to the metabolic reprogramming of many types of cancer cells, glucose is mainly catabolized by aerobic glycolysis in tumors, while the fate of Glutamine is completed at mitochondrial level where the enzyme Glutaminase converts Glutamine to Glutamate. Glutamine rewiring in cancer cells is heterogeneous. For example, Glutamate is converted to α-Ketoglutarate giving rise to a truncated form of Krebs cycle. This reprogrammed pathway leads to the production of ATP mainly at substrate level and regeneration of reducing equivalents needed for cells growth, redox balance, and metabolic energy. Few studies on hypothetical mitochondrial transporter for Glutamine are reported and indirect evidences suggested its presence. Pharmacological compounds able to inhibit Glutamine metabolism may represent novel drugs for cancer treatments. Interestingly, well acknowledged targets for drugs are the Glutamine transporters of plasma membrane and the key enzyme Glutaminase.

## Introduction

**Figure 1:**
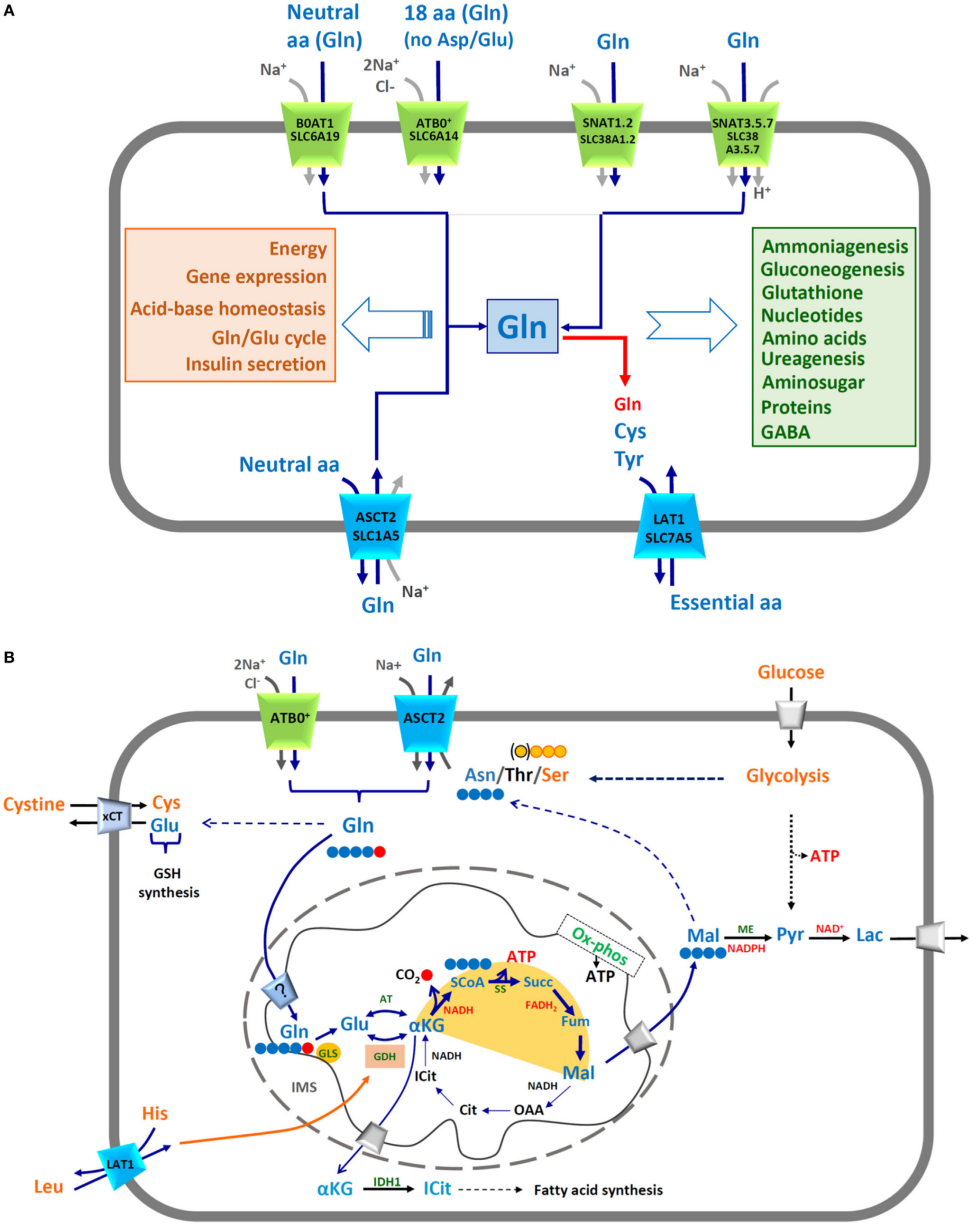
**(A)** Membrane transporters of glutamine and mechanisms of transport. The shape of the transporters reflects their asymmetry in membrane. Transporters are indicated by both conventional and SLC names. Different colors highlight different transport modes: in green symporters, in blue antiporters. Arrows represent direction of transported amino acids (blue) and ion (grey) fluxes; red arrow indicates possible Glutamine exit *via* LAT1 (SLC7A5). In the orange box, the list of cell pathways in which Glutamine is involved; in the light green box, the list of molecules synthesized from Glutamine. **(B)** Mitochondrial and cytosolic pathways responsible for energy production from Glutamine. In the scheme, Glutamine (Gln, blue) uptake occurs *via* membrane transporters ATB^0,+^ and ASCT2 through a sodium coupled process. The pathways are indicated as solid or dotted (in the case of multistep pathways) arrows (in blue those related to Glutamine, in black those involved in other pathways). Carbon atoms of Gln are depicted in blue–red filled circles; Gln enters mitochondria *via* an inner membrane transporter whose existence is still questionable (?): it could be a Glutamine or a Glutamate transporter depending on the actual sub-localization of Glutaminase enzyme (GLS). Carbon atom derived from Gln and released as CO_2_ is indicated in red, carbon skeleton of Malate and Asparagine (Asn) in blue, carbon skeletons of Serine (Ser) in orange circled in red and of Threonine (Thr) in orange circled in black. The truncated form of TCA is highlighted by a yellow hemicycle. ATP and reducing equivalent molecules produced by Glutamine metabolism are indicated in red. Leucine enters through LAT1 and allosterically regulates GDH in the orange box. Some metabolic pathways are indicated by names: GSH synthesis, fatty acid synthesis, Glycolysis, OX-phos. Membrane transporters of lactate and glucose in grey, xCT in light blue. Enzymes highlighted: GLS, Glutaminase; GDH, Glutamate dehydrogenase; AT, aminotransferases; SS, succinylCoA synthetase; ME, malic enzyme; IDH1, isocitrate dehydrogenase. Amino acids and other molecules involved in glutamine pathways (azure): Glu, Glutamate; α-KG, α-ketoglutarate; ICit, isocitrate; SCoA, succinyl coenzyme A; Succ, succinate; Fum, fumarate; Mal, malate; OAA, oxaloacetate; Cit, citrate; Pyr, pyruvate; Lac, lactate.

A conspicuous number of scientific reports clearly show that cancer is a metabolic disease (1–3). Metabolic reprogramming is driven by changes in expression of specific genes that allow cancer cells escaping control mechanisms active in healthy cells. The knowledge of these variations is relevant for designing novel and more specific pharmacological strategies. Therefore, many unknown or controversial aspects of cancer cell metabolism are object of active investigation. In this respect, mitochondria are crucial for cell survival and their features in cancer vary profoundly in terms of DNA content, electron chain functionality, and ATP production (4, 5). In this complex scenario, Glutamine is a key player since it is a versatile amino acid whose carbon skeleton is employed in different cell compartments for several purposes. Noteworthy, in physiological conditions as well, Glutamine is the most abundant amino acid in plasma, reaching a concentration of 0.8 mM and it can rise up to 40% of the total amino acids intracellular content (6). Glutamine is endogenously synthesized from α-Ketoglutarate, *via* Glutamate dehydrogenase and Glutamine synthetase. However, when cells are highly proliferative, the request of Glutamine increases and it has to be absorbed from external sources (7), making Glutamine a “conditionally essential” nutrient. Hence, some cancer cells are considered “glutamine addicted” because their growth and proliferation rates depended on availability of this amino acid (8, 9). Glutamine is engaged in different pathways, both cytosolic and mitochondrial, responsible for synthesis of many molecules (Figure 1A). Glutamine is also involved in other cell processes such as, Glutamine/Glutamate cycle in nervous tissue (Figure 1A) (10, 11). Glutamine ends its fate in mitochondria to be oxidized, producing ATP. Some aspects of the Glutamine transport and mitochondrial metabolism, which characterize cancer cells, will be dealt with. Noteworthy, Glutamine has been proposed to activate cell growth also independently from energy metabolism, by acting on signaling processes (11, 12).

## Glutamine Supply to Cancer Cells

The higher demand of glutamine by some cancer cells requires the action of membrane transporters with two essential features: (i) specificity for Glutamine and (ii) high transport capacity. Membrane transporters for amino acids are characterized by a broad specificity. In other words, the same transporter is able to recognize different amino acids with a redundancy that is typical of this class of proteins (13). In particular, Glutamine is recognized as substrate by some of the members of four different SLC families, which are clustered on the basis of phylogenetic analyses: SLC1, SLC6, SLC7, and SLC38 (14). Each transporter can be indicated by either the SLC or the old nomenclature (Figure 1). Even though the genetic and biochemical characterization of Glutamine transporters began several years ago, many unclear aspects are still existing especially in the frame of concerted action and regulation of the transporters and to their importance in Glutamine homeostasis under physiological (Figure 1A) and pathological conditions (13, 14). A remark is, however, very clear: some of the transporters sharing specificity for Glutamine are overexpressed in many tumors, i.e., ASCT2, ATB^0+^, and LAT1 (Table 1) (15–17); notwithstanding, not all of them are suitable for providing cells with high amount of this amino acid since they do not fulfill both the features above mentioned. A concise summary of the major players of Glutamine homeostasis is reported below together with an update on the most likely transport mechanisms underlying their role in cancer.

**Table 1:** ATB^0,+^, ASCT2, and LAT1-associated cancers.

SLC6A14 (ATB^0,+^)	SLC1A5 (ASCT2)	SLC7A5 (LAT1)	Reference
Prostate cancer	Prostate cancer	Prostate cancer	(14, 18–22)
Colorectal cancer	Colorectal cancer	Colorectal cancer	(14, 23)
	Hepato cell carcinoma	Hepato cell carcinoma	(14)
	Lung cancer	Lung cancer	(14, 24)
Breast cancer	Breast cancer	Breast cancer	(14, 18, 25–28)
	Neuroblastoma and glioma	Neuroblastoma and glioma	(14, 29)
	Endometrioid carcinoma	Endometrioid carcinoma	(14, 30, 31)
	Ovarian cancer	Ovarian cancer	(14, 32)
	Renal cell carcinoma	Renal cell carcinoma	(14, 33, 34)
Pancreatic and biliary tract cancer		Pancreatic and biliary tract cancer	(14, 35, 36)
	Gastric cancer	Gastric cancer	(14, 37–40)
		Pleural mesothelioma	(14)
Cervical cancer	Cervical cancer		(41, 42)
	Oral squamous cell carcinoma	Oral squamous cell carcinoma	(43–45)
		Thymic cancer	(46)
		Melanoma	(47)
		Leukemia	(48)

*List of cancer tissues in which ATB^0,+^, ASCT2, and/or LAT1 have been found overexpressed with related references*.

SLC1A5 is referred to as ASCT2, acronym standing for Alanine, Serine, Cysteine Transporter according to preliminary observations on substrate specificity (13). Recently, we showed that the actual preferred substrate is Glutamine and that Cysteine is not a substrate but, probably, a modulator of transport activity, in agreement with the previous reports describing a very low transport of Cysteine, if any (49, 50). The specificity of ASCT2 toward Glutamine correlates well with its overexpression in several human cancers (16, 51); to better explain its role in Glutamine addiction, many authors depicted ASCT2 as a Na^+^-dependent symporter of Glutamine, thus apparently fulfilling the two constraints above listed, i.e., specificity and high transport capacity (52–55). However, the proposed mechanistic model does not correlate with the actual transport mode of ASCT2 that is a Na^+^-dependent antiporter, according to both initial and more recent studies, including ours, which well clarify this aspect (16, 49, 56, 57) (Figure 1A). Therefore, at variance with the common view, the uptake of Glutamine, required by cancer cells, must be coupled to an opposite and quantitatively equal efflux of another neutral amino acid. Under a metabolic point of view, it is reasonable that the most probable exchanged amino acids are Asparagine, Threonine, or Serine; these, indeed, are high affinity substrates of ASCT2 (56) and the antiport with Glutamine will allow the net entry of 1–2 carbon atoms into the cell, which can be oxidized in the TCA to produce ATP (Figure 1B). This reaction is energetically favored by extracellular sodium gradient and membrane potential; the transporter is electrogenic due to net positive charge accumulation, as we recently highlighted (56). This “amino acid exchange” mechanism correlates well with the increased plasma concentration of Serine and Threonine, widely described in different cancers (58). Over the years, overexpression of ASCT2 has been associated also to another transporter of neutral amino acids, SLC7A5 referred to as LAT1 (59), as originally proposed by Fuchs and Bode (16). This protein is a Na^+^-independent obligatory antiporter and it has an heterodimeric structure, being associated to an ancillary protein named CD98 (SLC3A2) which, however, does not play any role in the intrinsic transport function (Figure 1A) (60). LAT1/CD98 heterodimer is broadly expressed and provides cells with essential amino acids, such as Leucine, in those body districts where these are required for cell growth. Indeed, strong genetic alterations of LAT1 in embryo are not compatible with life and very few are found in families characterized by some cases of Autism Spectrum Disorders, in which the metabolic damage is ascribed to altered supply/excessive loss of essential amino acids, in particular Histidine, to/from brain (61). LAT1 is greatly overexpressed in tumors where it has a role in signaling function (Table 1) (16, 51). Leucine, indeed, modulates the activity of one of the master cell growth regulators: mTOR (62). This protein kinase senses amino acid availability and it is particularly responsive to Leucine, Glutamine, and Arginine levels across lysosomes (62). In this respect, it is worth to note that LAT1, besides in plasma membranes, has also been found in lysosomes together with the “transceptor” SLC38A9 (63–65). Moreover, Leucine is a positive allosteric regulator of Glutamate dehydrogenase, which is responsible of Glutamine fate in mitochondria (17). For all the stated reasons, both LAT1 and ASCT2 can be considered eminent targets for drugs (51). However, the commonly proposed model in which Glutamine is taken up *via* ASCT2 to boost the transport cycle of LAT1, for massive entry of Leucine, is questionable. Indeed, as above described, ASCT2 is not a symporter, but an antiporter, and Glutamine is a poor substrate of LAT1 (60) (Figure 1). Thus, it is necessary to reconsider an integrated view of metabolism, which takes into account other membrane transporters. In particular, two members of SLC6 family are characterized by both specificity for Glutamine and high transport capacity and are involved in supplying it to cells in physiological and pathological conditions (Figure 1): SLC6A14 and SLC6A19 known as ATB^0,+^ and B^0^AT1, respectively (66). In the case of ATB^0,+^, Glutamine uptake has been proposed to be coupled with 2Na^+^ and 1Cl^−^ while, in the case of B^0^AT1, it is coupled to Na^+^ (Figure 1A). The transport cycle of the two proteins is electrogenic making ATB^0,+^ and B^0^AT1 high capacity transporters. Despite this, no involvement in cancer is reported for B^0^AT1, so far. Altered expression of this protein is described only in an inherited disease referred to as Hartnup disorder (67). On the contrary, a number of studies shows overexpression of ATB^0,+^ in human cancers (25, 51) (Table 1). Therefore, this protein can be considered one of the players in accomplishing metabolic needs of cancer cells and, hence, a druggable target (Figure 1B). However, at this stage, a plausible unified model, including ASCT2, LAT1, and ATB^0,+^ cannot be predicted because the study on biology of the last one is still in embryonic form. The only available information concerns its broad specificity and localization (66). Another family characterized by a sizable number of Glutamine transporters is the SLC38, which accounts for 11 members, the best known of which are described as Glutamine transporters coupled to Na^+^ or Na^+^/H^+^ fluxes (68) (Figure 1A). Wide proteomic/genomic data indicate that some of the SLC38 members are overexpressed in human cancers (69). Further studies are required to establish a direct role of these transporters in Glutamine supply and, hence, their possible consideration as drug targets. Noteworthy, an important advancement has been recently provided in the field of cell signaling linked to amino acid sensing with the discovery that SLC38A9 is a lysosomal transporter responsible for Glutamine and Arginine flux across lysosome with consequent activation of mTOR cascade (64, 65).

## Glutamine Metabolism in Mitochondria and the Still Unsolved Transport Issue

The relevance of Glutamine for energy production underlies a truncated form of TCA characterizing the mitochondrial metabolism of several type of cancers. In this pathway, the cycle is not completed and the carbon skeleton of Glutamine, entering the TCA as α-Ketoglutarate, escapes as Malate with production of ATP at substrate level in the reaction catalyzed by the Succinyl-CoA Synthetase. According to this pathway, one out of the five carbon atoms of Glutamine, is released as CO_2_ (Figure 1B). The four remaining carbon atoms of Glutamine are exported in cytosol as Malate that can give rise to different metabolic pathways. It can be converted into Pyruvate leading to NADPH production that can be used by fatty acid synthesis or other biosynthetic pathways (70). Pyruvate can, in turn, be transformed to Lactate, restoring NAD^+^ needed for anaerobic glycolysis and production of ATP (Figure 1B). This typical anaerobic pathway occurs even in the presence of adequate oxygen supply, according to the well-acknowledged Warburg hypothesis (16, 71, 72). Alternatively, Malate can enter four carbon atom molecules among which Asparagine, i.e., one of the substrates necessary for ASCT2 transport cycle (Figure 1B). In this case, Malate is converted into oxaloacetate *via* malate dehydrogenase and then, to aspartate *via* aspartate aminotransferase (resumed by the dotted arrow of Figure 1B). The alternative efflux substrate of ASCT2, Serine can derive from glucose *via* a three enzymes pathway, i.e., phosphoglycerate dehydrogenase, phosphoserine aminotransferase, and phosphoserine phosphatase (resumed by the dotted arrow of Figure 1B). Noteworthy, the reaction catalyzed by the second enzyme (aminotransferase) requires Glutamate, which in turn derives from Glutamine. On the other hand, Threonine, which could be an efflux substrate of ASCT2 as well, is an essential amino acid; thus, it should derive from import through other transporters or, hypothetically, from protein degradation. Moreover, Glutamine skeleton can also fuel fatty acid synthesis in cytosol by reductive carboxylation of α-Ketoglutarate, exported from mitochondria, to isocitrate through the action of a cytosolic isoform of IDH (Figure 1B). This is a non-conventional reaction for producing citrate, occurring in cells that undergo metabolic switch (70, 73, 74). Glutamine is involved also in ROS metabolism, which is another crucial point for cancer development and progression (75). Cancer cells, indeed, need to keep the production of ROS under strict control *via* mechanisms involving both enhanced glutathione (Glutamate-Glycine-Cysteine—GSH) synthesis and decreased respiratory chain activity. Glutamate needed for GSH synthesis derives, under these conditions, from Glutamine (Figure 1B) (76). Cysteine is taken up by cells *via* the Glutamate/Cystine transporter xCT (SLC7A11), which has been found overexpressed in several cancers and is responsible for a novel way of cell death called ferroptosis (77). Thus, Glutamine withdrawal can have dramatic effects on cancer cell metabolism (75, 78). Despite the described importance of Glutamine in mitochondrial metabolism, the network of proteins involved in its flux to mitochondrial matrix is still underneath. Several efforts have been made to shed light on two mitochondrial molecular entities, which are still mysterious: the enzyme Glutaminase and the mitochondrial transporter for Glutamine (Figure 1B). Glutaminase is produced by two different genes: GLS1 and GLS2. The first one is known as kidney-type Glutaminase and is ubiquitously expressed. The GLS2 gene is known as liver-type glutaminase (LGA) and is mainly expressed in liver. The GLS1 type is subjected to alternative splicing producing a full isoform and a truncated one, which differs for its C-ter region and is known as Glutaminase C (79). These two isoforms have been found overexpressed in different cancers, in line with the increased metabolic demand of mitochondrial Glutamine (80). The importance of this enzyme in the fate of Glutamine is testified by a number of different pathways involved in its regulation among which, c-Myc, whose action is exerted through inhibition of a microRNA, miRNA-23a that results in increased GLS1 expression and, then, activity (81). Under a pharmacological point of view, Glutaminase represents an important target for anticancer therapy (82). However, the sub-localization of mitochondrial Glutaminase is not yet defined and, as a consequence, the need of a mitochondrial Glutamine transporter. In fact, if Glutaminase faces the intermembrane space, here, releases Glutamate then, a Glutamate transporter, not a Glutamine one, is required to allow entry of Glutamate in the TCA. On the contrary, if Glutaminase faces the intra-mitochondrial matrix, then a Glutamine transporter is necessary to allow Glutamine reaching the substrate active site of Glutaminase (Figure 1B). Biochemical data, even though indirect, agree with the second hypothesis and, hence, with the existence of a Glutamine transporter (Figure 1B) whose molecular identity is not yet revealed (82–86). We have conducted *in silico* analyses aligning a putative Glutamine binding motif with members of the mitochondrial transporter SLC25 family: the best score was obtained for three orphan SLC25 members resulting as possible mitochondrial Glutamine transporters (11).

## Glutamine Metabolism as Target for Drugs

The complex network of enzymes/transporters involved in Glutamine metabolism explains the plethora of drug interventions to specifically target cancer cells. A big challenge is the metabolic adaptation of cancer cells that can survive also under stress conditions, such as Glutamine withdrawal (87, 88). Last, but not less important, is the great diversity of cancers; thus, it is not surprising that therapeutic interventions needs to be specifically designed. Being Glutamine a key player in multiple pathways, the most important makers of its fate represent potential crossroad for cancer therapy. In particular, inhibitors of the key enzyme Glutaminase have been designed over the years (7, 82) and their studies are at a more advanced stage, being Glutaminase a soluble protein, i.e., easier to handle also *in vitro*. Interestingly, murine Glutaminase 3D structure has been obtained (pdb 4JKT) and, very recently, the human one has been deposited in the database (pdb 5UQE), as well. Some inhibitors showed very good results in *in vitro* models of human cancers and few of them were promising in preclinical studies. In particular, one synthetic compound, i.e., CD-839 reached clinical trials due to its ability to block tumor growth *in vitro, in vivo*, and in mouse models (89). The main challenges with respect to Glutaminase inhibitors are the presence of more than one isoform of GLS and the still unsolved issue of subcellular localization that can hamper the drug availability. The scenario around membrane transporters is even more complex. In fact, their relevance in pharmacology is obvious and relies on two main aspects: membrane proteins can be (i) target of designed drugs and/or (ii) responsible for drug traffic across membranes and, thus, for drug disposition. This second aspect is still not fully considered by the scientific community that did not include any transporter for amino acids in the list of the International Transporter Consortium for drug–transporter interactions (90). The frontiers of drug design are based on *in silico* models that, on the one hand, reduce the number of experimental analysis to be conducted; on the other hand, if the 3D model of the protein is obtained by homology, predictions may be uncertain. This circumstance, in the case of membrane transporter, occurs quite often because few 3D structures are available so far. The well-documented overexpression of some membrane transporters, above described (see Glutamine Supply to Cancer Cells; Table 1), boosted the research of potent and specific inhibitors; in particular, several reports dealt with the identification of inhibitors for ASCT2 (91) and LAT1 (92) *via* bioinformatics. The initial approach, attempted over the years, has been that of designing substrate analogs-based drugs to block either ASCT2 or LAT1 transport activities (93, 94). However, all the discovered molecules exhibited relatively low affinities and, hence, low effects on reducing cancer cell viability. The pitfalls of this strategy are explained by the frame schematically depicted in Figure 1A; in fact, membrane transporters of amino acids are poly-specific meaning that natural substrates can displace a hypothetical substrate-based drug. These compounds, in fact, interact only transiently with the target protein leading to scarce effects. In the recent years, we have exploited a combined approach of bioinformatics, *in silico* screening and biochemical assays using the *in vitro* experimental model of proteoliposomes in order to identify covalent inhibitors for both ASCT2 and LAT1. Being irreversible, covalent inhibitors should be in principle, more efficient in chemically knocking-out the transporters. This strategy has the advantage of facilitating the compound screening studying the effects on the sole target protein, without interferences deriving from other systems present in the whole cells (95). Then, we identified potent covalent inhibitors of the rat ASCT2 (96). Soon after, we obtained also a set of covalent inhibitors of human LAT1 with the highest affinity so far described (97). LAT1, as mentioned above, even if is probably not directly linked to Glutamine uptake in cancer cells, is responsible for providing essential amino acids, among which Leucine (see Glutamine Supply to Cancer Cells). Test in intact cells showed that the compounds were also able to impair viability of cancer cells.

## Author Contributions

MS and CI wrote the manuscript and designed the figures. MG, LP, and LC contributed to revision of the manuscript, figures, and bibliography.

## Conflict of Interest Statement

The authors declare that the research was conducted in the absence of any commercial or financial relationships that could be construed as a potential conflict of interest.
